# Cooling history and emplacement of a pyroxenitic lava as proxy for understanding Martian lava flows

**DOI:** 10.1038/s41598-019-53142-0

**Published:** 2019-11-19

**Authors:** Mara Murri, Maria C. Domeneghetti, Anna M. Fioretti, Fabrizio Nestola, Francesco Vetere, Diego Perugini, Alessandro Pisello, Manuele Faccenda, Matteo Alvaro

**Affiliations:** 1 Department of Earth and Environmental Sciences, University of Pavia, Via A. Ferrata, 1, 27100 Pavia, Italy; 2 Istituto di Geoscienze e Georisorse CNR, Padova, Italy; 3 Department of Geosciences, University of Padova, Via G. Gradenigo 6, 35131 Padova, Italy; 4 Department of Physics and Geology, University of Perugia, piazza Università 1, 06123 Perugia, Italy; 5 Institute of Mineralogy, Leibniz Universität Hannover, Callinstr. 3, D-30167 Hannover, Germany

**Keywords:** Mineralogy, Petrology, Volcanology

## Abstract

Terrestrial analogues are often investigated to get insights into the geological processes occurring on other planetary bodies. Due to its thickness and petrological similarities, the pyroxenitic layer of the 120m-thick magmatic pile Theo’s Flow (Archean Abitibi greenstone belt Ontario, Canada), has always been regarded as the terrestrial analogue for Martian nakhlites. However, its origin and cooling history and, as a consequence those of nakhlites, have always been a matter of vigorous debate. Did this lava flow originate from a single magmatic event similar to those supposed to occur on Mars or do the different units derive from multiple eruptions? We demonstrate, by a combination of geothermometric constraints on augite single crystals and numerical simulations, that Theo’s Flow has been formed by multiple magma emplacements that occurred at different times. This discovery supports the idea that the enormous lava flows with similar compositions observed on Mars could be the result of a process where low viscosity lavas are emplaced during multiple eruptions. This has profound implications for understanding the multiscale mechanisms of lava flow emplacement on Earth and other planetary bodies.

## Introduction

## Terrestrial Lava Flows as Analogue for Martian Lavas: The Case of Theo’s Flow

Investigation of ancient lava flows provides unprecedented insights for the understanding of eruptive activity occurring on both the Earth’s surface and on other planetary bodies. An ancient lava flow such as Theo’s Flow, due to its thickness and lithology of the stratigraphic layers, represents the perfect candidate that has been used in the past 20 years as a terrestrial analogue for thick lava flows erupted on Mars.

Theo’s Flow is a 120 m-thick magmatic pile located in the mafic and ultramafic region of the Archean Abitibi greenstone belt (Munro Township, Ontario)^1^ that was affected by metamorphic alteration under greenschist facies conditions (e.g. <500 °C). However, the well-preserved textural relationships and absence of evidence of alteration of the augites within the flow suggest that they did not suffer either a resetting of their chemical signatures or metamorphic alteration^2^. The entire magmatic pile contains the following lithologies (bottom to top): peridotite (~9 m), pyroxenite (~60 m), gabbro together with a pegmatitic pyroxenite (~40 m) and hyaloclastite (~10 m thick). The top quenched margin of the hyaloclastite is a breccia^3^ that together with pillow basalts and pyroclastites^4^ suggests that subaerial setting followed by a submarine environment characterized the origin and evolution of Theo’s Flow. In particular, based on detailed petrography and geochemistry, the pyroxenite layer has always been regarded as the best terrestrial analogue of nakhlites^2,5^. This lava flow dates to ~ 2.7 Ga, based on ages determined on nearby komatiites^6^. The best outcrop of Theo’s Flow is in the direction of the flow^3^ and, as showed in Lentz *et al*.^2^, it is comprised of a magmatic pile of different lithologic units.

Based on detailed petrological and stratigraphic analyses Lentz *et al*.^2,5^ concluded that the lithologic diversity observed in Theo’s Flow was the result of a differentiation process of a single, thick magma pulse, rather than being generated by the eruption of multiple magmatic batches with distinct composition. This conclusion was based on the absence of sharp boundaries between adjacent units and on the progressive compositional changes observed in mineral and bulk-rock compositions. According to these authors^2^, even if this hypothesis was further supported by the matching between the average composition of the whole magmatic pile with that of the quenched hyaloclastite breccia at the top, the latter being considered as having the composition of the primary magma, “the exact nature of the differentiation process remains uncertain”. Therefore, the origin of Theo’s Flow and its cooling history have been extensively debated^2,5,7,8^ mostly because the original assumption of Lentz *et al*.^2^ that the 120 m of lava have been emplaced as a consequence of one single eruptive event. This would be a rather unusual phenomenon on planet Earth but is commonly accepted for Mars because, considering measurements made on imagery of actual Martian flows^9–13^, thicknesses of the order of 120 m seem reasonable.

However, several authors^14–16^ raised concerns about some inconsistencies between the cooling rate and temperature estimates obtained from petrographic/textural evidence (fast cooling rates 3–6 °C/h) against those from geothermometric data (slow cooling rates 0.1 °C/h). Because of these contrasting results Domeneghetti *et al*.^16^ and then Alvaro *et al*.^7^ proposed for nakhlites and Theo’s Flow, respectively, a mechanism of emplacement with subsequent injections blanketing one another, but this hypothesis has never been tested. Given the analogy with Martian lava flows, the mechanism and timing of differentiation of Theo’s Flow deserves further investigation to shed light on these geological processes on Mars.

## The Emplacement of Theo’s Flow: a Multi-Eruption Scenario

If we follow the idea that Theo’s Flow was emplaced into a pre-existing V shape topography (valley), a possible scenario for the emplacement mechanism could be related to effusive submarine eruptions as suggested in the literature^2^. Magma–water interactions can occur deep within the Earth or near the surface as possibly happened for the Theo’s Flow. In such a case, convective cooling by water should be considered as the dominant cooling mechanism because it is more efficient than convective, radiative, and conductive cooling observed on the Earth surface^17,18^. Fink and Griffiths^19^ and more recently Gregg^18^ proposed that the convective heat flux from the surface of a submarine lava flow leads to an almost immediate cooling of magma. As an example, after 1 s, a basalt erupting at 1423 K passes through the solidus and glass transition temperature at a depth of 2 mm and 0.5 mm, respectively. The rupture of crust is a reasonable process during surface eruption^20^ where the new and fast-formed crust can incur in rupture from which new blob of magma will come out. Active pillow lava lobe inflating on transverse and radial cracks with high-effusion-rate are plausible events for flows moving down to a steep slope. As an example, inflated Pahoehoe flows can display large inflation structures such as plateaus and cover several hundreds of square meters as observed in Iceland, and the inflation height can reach a maximum of 20 m in the case of submarine or subaqueous flows^20^. Since Theo’s Flow is described as a120 m thick lava flow this would require superimposed flows each of the order of 10–50 m thick closely emplaced in time. Finally, the study of lava flows in submarine environments requires that the effective gravity force acting on the flow is considered. Due to the lower density difference between the lava and the water it is smaller in water than on Earth surface^19^. The reduced gravity (g’) is given by:

1 $$g^{\prime} =\frac{g\,(\rho -\rho w)}{\rho }$$

and it takes into account *ρ* (the lava density) and density of the surrounding seawater *ρw*. With a lava density of 2600 kg m^−3^, if we consider a shallow subaqueous environment where density of water is close to 1000 kg m^−3^ and ambient water temperature ranges from 273 to 283 K, the effective gravity is 6.03 ms^−2^. Whereas, with the same lava density, at 1000 m deep and a temperature of 273 K where seawater has a density of 1046 kg m^−3^, the effective gravity is 5.85 ms^−2^. This implies that the buoyancy force countering the effect of the gravity is significantly higher in water and this greatly increases the ability of lava to inflate and also increases the thickness of the lava flow by about 30%^20,21^. Considering that in subaqueous environments the height of flows is on the order of 10 m we should therefore explore the possible scenario already proposed^7,16^ of superimposed lava flows rather than one single-flow unit.

Thermal constraints on slowly cooled paleo lava flows cannot rely upon conventional chemical geothermometry based on intercrystalline cation exchange because at low temperature (below 600–700 °C) long-range diffusion is kinetically hindered. Conversely, the short range of intracrystalline exchange reaction can proceed to much lower temperatures (i.e. about 500 °C, see^22,23^ for further details) thus allowing it to be used to estimate the cooling history for complex magma bodies that may have interacted with one another.

## Experimental Closure Temperature and Cooling Rates

To understand the magmatic evolution and the cooling history of the whole of Theo’s Flow we applied a newly-determined geothermometric calibration^24^ of the Fe^2+^-Mg intracrystalline exchange reaction between *M*1 and *M*2 sites in augite crystals^7,22–30^ to determine the closure temperature (*T*_*C*_) and cooling rates for each lithological layer in the Theo’s Flow pile.

**Figure 1:**
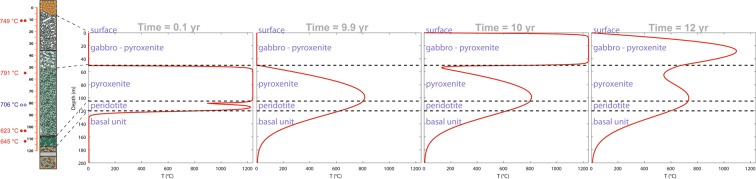
Lithostratigraphic column for the lava pile on the left with closure temperatures determined for all the samples in this study (red dots) and previously^16^ (blue open circles). Thermal evolution over time for each layer of the Theo’s Flow lava pile. The peridotite and pyroxenite emplacements are approximately coeval and they are followed by the gabbro – pyroxenite emplacement 10 years later.

We selected 6 augite single crystals from 4 small chips (5 to 10 grams) sampled at four different stratigraphic depths below the cooling surface of Theo’s Flow (red dots in Fig. 1). One crystal has been selected from the peridotite (TSC 3.9) and five from the pyroxenite layers (TSC 3.12_1 TSC 3.12_2; TSC 3.22; TSC 3.31_1; TSC 3.31_2) sampled at different depths (see Fig. 1). These results have been combined with those obtained by Domeneghetti *et al*.^16^ on two more crystals from the pyroxenite layer at an intermediate depth (see blue open circles in Fig. 1). All of the selected clinopyroxenes have Fs contents [with Fs = 100 * ΣFe/(ΣFe + Mg + Ca) where ΣFe = Fe^2+^ + Fe^3+^ + Mn] within the compositional range of Fs_9_ to Fs_24_ previously reported by Murri *et al*.^24^. High-resolution microfocus (beam size 120 μm) single-crystal X-ray diffraction measurements [MoKα (λ = 0.71073 Å) operating at 50 kV and 0.8 mA (power = 40 W)] have been carried out on each crystal. The collected intensity data (Table S1) have been used for structure refinements to determine the cation site occupancies (Table S2) adopting the results from electron microprobe analyses (i.e. EMPA) to constrain the composition^16^. Full compositional data of the selected six augite crystals obtained by electron microprobe are reported in the Supplementary Material (Table S3).

The Fe^2+^-Mg ordering state was obtained from the site populations in order to calculate the closure temperature by means of the intracrystalline distribution coefficient (*k*_D_), using the expression^29^ *k*_D_ = [(Fe^2+^_M1_) (Mg_M2_)/(Fe^2+^_M2_)(Mg_M1_)]. The relationship between the distribution coefficient (*k*_D_) and *T*_C_ is usually expressed using calibration equations of the *lnk*_D_ as a function of 1/*T*(K).

For our augite samples the calibration equation^24^, reported in Eq. 2, was used because it produces reliable results on samples with compositions ranging between Fs_9_ and Fs_24_ as demonstrated from the investigation of synthetic samples^23^. In particular, the equation was obtained using data from^24^ Theo’s Flow augite (TS7 N.16, Fs_9_) together with those from augites^7^ from the Miller Range nakhlite sample (MIL 03346, Fs_24_).

2 $$ln{k}_{D}=-\,4040\,(\,\pm \,180)/T(K)+1.12\,(\,\pm \,0.17)\,({{\rm{R}}}^{2}=0.988)$$

The resulting *T*_C_ are reported in Table 1 and in Fig. 1 (where an average *T*_C_ is reported for levels where two crystals were sampled). They show a thermal gradient of about 170 °C across the pyroxenite unit. Furthermore, the maximum recorded *T*_C_ value is 791(15) °C at 65 m depth and towards the top of the flow the closure temperatures drop to values of 734 (19) °C and 764 (20) °C. In particular, the closure temperature of 791 °C (top of the pyroxenite unit) indicates a relative fast cooling rate with respect to all the other measured temperatures suggesting that, at the time of its eruption, no material was present above the pyroxenite and it was cooled down at the contact with the atmosphere (subaerial, submarine environment). Therefore, this evidence coupled with the observed thermal gradients and measured closure temperatures seems to point towards a multiple-eruption scenario rather than to support the hypothesis^2^ of one single magma emplacement differentiated *in-situ*. In this new scenario the different composition of the lithologic units could be therefore be simply ascribed to an initial differentiation process in the magma chamber prior to eruption.

**Table 1:** *Cooling rates estimated by using the model from^22^, ^†^cooling rates from the modeled scenario with multiple injections and those from a unique magma emplacement^‡^.

Units	Gabbro-Pyrox.	Top Pyrox.	Middle Pyrox.	Low Pyrox.	Peridotite
*T*_C_ (°C)	749	791	706.5	622	645
CR (°C/h)*	0.0463	0.2326	0.0086	0.0028	0.0056
CR (°C/h)^†^	0.0438	0.1707	0.0058	0.0041	0.0055
CR (°C/h)^‡^	0.0431	0.0018	0.0015	0.0014	0.0019

## Reconstructed Thermal History For Theo’s Flow

To further test this new hypothesis, we carried out numerical simulations to assess the possibility of different magma emplacements blanketed by subsequent events of different durations as previously suggested^7,16^. This model couples the closure temperature with the cooling rate determined on each single crystal, providing the timing for each subsequent lava flow emplacement.

**Figure 2:**
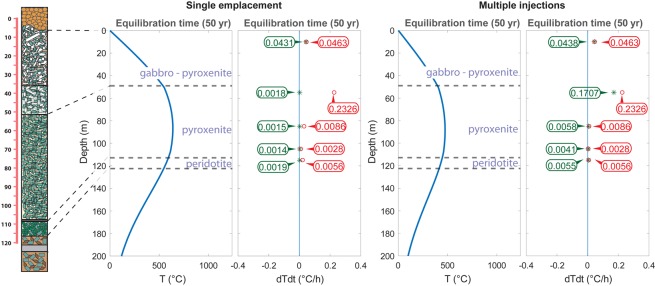
Comparison between the cooling rates determined on our samples following the approach by^22^ (red labels) and those calculated from our 1D model (green labels) comparing the single emplacement vs multiple subsequent emplacements.

We solved the 1D heat diffusion equation with the finite difference method in a 300 m thick vertical profile considering the variation of physical properties due to the different lithologic units (see Table S4 and Fig. 2). We imposed a surface temperature (*T*_surf_ = 0 °C) in the uppermost 120 m of the profile to simulate the initial presence of air/water, whereas we have computed the temperature according to a steady state geotherm for the basement (120–300 m). The emplacement of a single lava flow is modelled by increasing the temperature instantaneously from T_surf_ to T_magma_ (T_magma_ = 1240 °C^2^^,^^31^). In order to study the influence of the timing of the eruption on the cooling rates we performed several simulations by varying the eruption time sequence. Average cooling rates have been estimated as:

3 $$CR\,(^\circ C/h)=({T}_{magma}-{T}_{closure})/({t}_{closure}-{t}_{emplacement})$$

where T is the temperature in °C and t is the time in hours. Figure 2 and Table 1 compare the cooling rates calculated using the model from^22^ (see Alvaro *et al*.^7^ for further details) with those determined from the modeled scenarios.

In summary, the scenario which best reproduces the cooling rates inferred from the intra-crystalline distributions that we measured in augites is that for which the pyroxenite lava flow occurs immediately after the peridotite (i.e. 0.1 year later). This time span is sufficient for the pyroxenite to blanket the peridotite and slow its cooling rate according to the measured closure temperature. The gabbro-pyroxenite lava flow occurs in a later stage with a minimum interval of 10 years after the emplacement of the pyroxenite.

The late stage-emplacement of the gabbro-pyroxenite unit allows the pyroxenite beneath to cool down sufficiently fast in agreement with our closure temperature (791 °C) estimate without invoking any reset of the Fe-Mg ordering degree (i.e. no chemical re-opening of the system) as we would have considered for the hypothesis of a single stage emplacement differentiated *in-situ*. The latter hypothesis can be excluded even more considering the low viscosity of the pyroxenite measured by Vetere *et al*.^31^ that undergoing fast cooling would not have had time to differentiate after emplacement but could be consistent with differentiation in a magma chamber. Furthermore, the closure temperatures determined in this work coupled with the low viscosity data by Vetere *et al*.^31^ on the same composition, also explain the high (120 m) thickness for these lavas on Earth (e.g. Ontario lava flows). The only contrasting evidence that poses against the multistage event could be the absence of the hyaloclastite at the top of each layer and the gradational contacts between the subsequent lithologic unit.

However, even in the multiple stage emplacement scenario, we could expect gradational contacts between the peridotite and the pyroxenite since they are almost coeval. Also between the pyroxenite and the gabbro a gradational contact can be expected because of the blanketing process in which the gabbro above the pyroxenite unit reheated its topmost part (up to 500–600 °C) without re-opening the Fe-Mg system in the augites that would require much higher T (*T*_*C*_ = 791 °C, see Fig. 1). The absence of the hyaloclastite can simply be explained by its mechanical erosion^32,33^.

## Conclusions

Because of its similarity with Martian lava flows Theo’s Flow has always been regarded as the analogue to be used for interpreting magmatic processes on Mars. The 120 m-thick Theo’s lava pile has always been considered the result of one single eruptive event, a rather unusual phenomenon on Earth but commonly accepted for Mars. However, the inconsistencies between cooling rate and temperature estimates from petrographic/textural evidence (fast cooling rates 3–6 °C/h) and from geothermometric data (slow cooling rates 0.1 °C/h) led several authors^14–16^ to question the one-stage emplacement process in search for alternative scenarios.

To unravel this extensive debate, we used a combination of temperature constraints on single augite crystals and numerical simulations to demonstrate that Theo’s Flow has been formed by multiple magma emplacements at different times. We can therefore summarize this new hypothesis for the thermal history of Theo’s Flow as follows: in the Archean age, the erupted differentiated lavas flowed at very high temperatures in a turbulent regime^31^. Because of their low viscosity, these lavas flowed for considerable distances until they were trapped by topography. The first emplacements, almost coeval, were those of the peridotite and pyroxenite in a subaerial or subaqueous environment and then the gabbro flow mechanically-eroded^31–33^ the hyaloclastite at the top of the pyroxenite, thus leaving as the only preserved hyaloclastite quenched breccia the one on the top of the whole pile.

This discovery supports the idea that the enormous lava flows with similar composition found on Mars could be the result of a process where low viscosity lavas^31^ are emplaced during multiple eruptions. Moreover, Udry and Day^34^ recently hypothesized a similar scenario for nakhlites and chassignites demonstrating that these lithologies were emplaced close or onto the surface of Mars as multiple flows. The proposed process explains at the same time the fast cooling rates recorded by the textures of the rock and the low closure temperatures recorded by the single minerals (e.g. intracrystalline exchange in pyroxenes) for which the subsequent magma emplacement would then re-equilibrate the system at much lower temperatures similar to those expected for high thicknesses single lava pile, at least for the peridotite-pyroxenite transition. This has profound implications for our understanding of multiscale mechanisms for lava flow emplacements on Earth and other planetary bodies.

## Methods

### Single-crystal X-ray diffraction and data reduction

X-ray diffraction experiments were carried out on each crystal using a diffractometer in the Department of Geosciences at the University of Padova^35^. The instrument consists of a Rigaku-Oxford Diffraction Supernova kappa-geometry goniometer equipped with an X-ray micro-source assembled with a Pilatus 200 K Dectris detector. The micro-X-ray source, MoKα (λ = 0.71073 Å) operates at 50 kV and 0.8 mA (power = 40 W). The micro source ensures a brilliance at least ten times higher than conventional sealed X-ray tubes and a beam spot of ∼0.120 mm. At the same time, the Pilatus 200 K detector ensures a very high sensitivity and negligible noise. Data collections have been performed using the Crysalis software (Rigaku-Oxford-Diffraction©) package. Data reduction, including intensity integration together with background and Lorentz-polarization corrections, have been carried out within the Crysalis package. The unit-cell parameters, the discrepancy indices *R*_int_, *R*_*all*_, *R*_*w*_ on all the *F*_o_^2^ and the goodness of fit (*S*) of the structure refinements with chemical constraints for the six crystals are reported in Table S1.

### Electron microprobe analyses (EMPA)

Major and minor elements were analyzed with a Cameca-SX50 electron microprobe with a fine-focused beam (1 μm diameter) operating in wavelength-dispersive (WDS) mode. Operating conditions were 15 kV accelerating voltage and 15 nA beam current; counting times were 20 s on the peaks and 20 s on the background. The following synthetic endmember mineral standards were used: diopside for Mg, ferrosilite for Fe, wollastonite for Si and Ca, chromite for Cr, corundum for Al, MnTiO_3_ for Mn and Ti, and a natural albite (Amelia albite) for Na. X-ray counts were converted into oxide weight percentages using the PAP correction program (as in Fioretti *et al*.^36^). Analyses are precise to within 1% for major elements and 3–5% for minor elements. The results of the chemical analysis are reported in Table S2. The crystal chemical formula was calculated on the basis of six oxygen atoms^37^. Only those spot analyses with total cation contents of 4.000 ± 0.005 atoms on the basis of six oxygen atoms and charge balance 3^[4]^ Al + Na − 3^[6]^Al − 4Ti − 3Cr − 3Fe^3+^ ≤|0.005| were selected and averaged. The Fe^3+^ content was calculated by stoichiometry^38^.

### Structure refinements

The observed F_o_^2^ values were then treated with a full-matrix least-squares refinement in the *C*2/*c* space group by using the SHELX-97 program^39^, starting from the atomic coordinates of sample TS7 N.2^16^ and taking into account the *M*21 and O2B1 split sites^40^, that were refined with isotropic displacement parameters. The atomic scattering curves were taken from *the International Tables for X-ray Crystallography*^41^. Neutral versus ionized scattering factors were refined for all sites not involved in chemical substitutions to ensure charge neutrality^42–44^. Complete ionization was assumed for Mg and Fe in the *M*1 site, for Ca and Mg in the *M*2 site and for Fe in the *M*21 site. Individual weights and the weighting scheme suggested by the program were used. The extinction correction was applied with the procedures of the SHELX-97 program. Table S1 reports the mean atomic numbers (m.a.n.) in electrons per formula unit (e.p.f.u.) at the crystallographic sites (*M*1, *M*2, *M*21) obtained when the structure refinement reached convergence, before introducing the chemical constraints. For all samples the calculated mean atomic numbers from the refinements unconstrained agree within less than 1 standard deviation with the values of electrons per formula unit (e.p.f.u.) calculated from the EMPA (Table S2). Therefore, this enabled us to use the results from the EMPA as chemical constraints for the structural refinements, following the procedure and taking into account the same constraints as in^16^ assuming 1 standard deviation as the error, in order to determine the clinopyroxene site distribution. The site populations obtained from the structural refinements with chemical constraints and the distribution coefficients (*k*_D_) with relative propagated errors are reported in Table S3.

## Supplementary information

Supporting Information
