# Supplementary material for: Cooling history and emplacement of a pyroxenitic lava as proxy for understanding Martian lava flows
Source: Sci Rep. 2019 Nov 19;9:17051. doi: 10.1038/s41598-019-53142-0 (PMC6864032; doi:10.1038/s41598-019-53142-0)
Supplement: Supplementary file 1 — Supporting Information [file 41598_2019_53142_MOESM1_ESM.pdf]

Supporting Information

Cooling history and emplacement of a pyroxenitic lava as proxy for understanding Martian lava flows

Mara Murri[1], Maria C. Domeneghetti[1], Anna M. Fioretti[2], Fabrizio Nestola[3], Francesco Vetere[4,5], Diego Perugini[4], Alessandro Pisello[4], Manuele Faccenda[3], Matteo Alvaro[1*]

[1]Department of Earth and Environmental Sciences, University of Pavia, Via A. Ferrata, 1 27100 Pavia, Italy
[2]Istituto di Geoscienze e Georisorse CNR, Padova, Italy
[3]Department of Geosciences, University of Padova, Via G. Gradenigo 6, 35131 Padova, Italy
[4]Department of Physics and Geology, University of Perugia, piazza Università 1, 06123 Perugia, Italy.
[5]Institute of Mineralogy, Leibniz Universität Hannover, Callinstr. 3, D-30167, Hannover, Germany.

**Corresponding author:* matteo.alvaro@unipv.it

Contents

Table S1 – Unit cell parameters ....
Table S2 – Site populations ....
Table S3 – Chemical Analyses (EMPA) ....
Table S4 – Physical properties of the lithological units ....

Table S1 – Unit cell parameters

Table S1. Unit cell parameters for clinopyroxenes TSC 3.9, 3.12_1, 3.12_2, 3.22, 3.31_1 and 3.31_2 and information on data collection and structural refinement.

	**CPX 3.9**	**CPX 3.12_1**	**CPX 3.12_2**	**CPX 3.22_1**	**CPX 3.31_1**	**CPX 3.31_2**
a (Å)	9.74318 (17)	9.74290 (14)	9.7434 (2)	9.74870 (18)	9.74221 (12)	9.74248 (13)
b (Å)	8.92390 (13)	8.92082 (11)	8.92133 (19)	8.92808 (13)	8.92235 (10)	8.92335 (10)
c (Å)	5.25403 (11)	5.25360 (8)	5.25381 (14)	5.2566 (1)	5.25425 (7)	5.25463 (8)
β(°)	106.233 (2)	106.2359 (16)	106.174 (3)	106.2034 (19)	106.3216 (13)	106.2884 (15)
V (Å^3)	438.61 (1)	438.40 (1)	438.61 (2)	439.35 (1)	438.31 (1)	438.48 (1)
θ_{max} - θ_{min} (°)	57-3.2	56.6-3.2	56.6-3.2	56.6-3.2	56.6-3.2	56.6-3.2
refl.no	27691	21124	31302	31261	29141	29352
I_{ind}	3011	2977	2988	2990	2990	2981
R_{int}	0.031	0.022	0.077	0.041	0.026	0.027
R_{all}	0.018	0.016	0.155	0.021	0.018	0.015
R_w	0.041	0.039	0.423	0.05	0.043	0.039
S	1.04	1.13	2.08	1.06	1.18	1.1
m.a.n. M1	13.51 (1)	13.35 (1)	13.38 (8)	14.02 (1)	13.68 (1)	13.58 (1)
m.a.n. (M2+M21)	20 (3)	19.89 (3)	20.05 (8)	20.16 (3)	20.05 (4)	20.07 (3)
m.a.n. (M1+M2+M21)	33.51 (3)	33.24 (4)	33.43 (11)	34.18 (3)	33.72 (4)	33.65 (4)

Standard deviations are given in parentheses. *refl.no.* is the number of unique reflections; I_{ind} is the number of independent reflections used for structure refinement; $R_{int} = \Sigma \mid F_o^2 - F_o^2(\text{mean}) \mid / \Sigma [F_o^2]$ where F_o and F_c are the observed and calculated structure factors; $R_{all} = \Sigma \mid \mid F_o^2 \mid - \mid F_c^2 \mid \mid / \Sigma [F_o^2]$; $R_w = \{\Sigma [w(F_o^2 - F_c^2)^2] / \Sigma [w(F_o^2)^2]\}^{1/2}$; $S = [\Sigma [w(F_o^2 - F_c^2)^2]/(n-p)]^{0.5}$, where *n* is the number of reflections and *p* is the total number of parameters refined. [(a)] m.a.n. is the mean atomic number (in electrons per formula unit) before introducing the chemical constraints. Crystal system monoclinic *C*2/*c*; radiation type Mo*K*α.

Table S2 – Site populations

Table S2. Site population for clinopyroxenes TSC 3.9, 3.12_1, 3.12_2, 3.22, 3.31_1 and 3.31_2 obtained from structure refinements using chemical constraints.

		**CPX 3.9**		**CPX 3.12_1**		**CPX 3.12_2**		**CPX 3.22**		**CPX 3.31_1**		**CPX 3.31_2**	
Site													
T	Si	1.948		1.952		1.952		1.971		1.967		1.971	
	Al	0.052		0.048		0.048		0.029		0.033		0.029	
M1	Mg	0.885	(1)	0.897	(1)	0.895	(3)	0.832	(3)	0.848	(3)	0.855	(3)
	Fe	0.050	(2)	0.042	(2)	0.044	(4)	0.130	(3)	0.105	(5)	0.102	(5)
	Fe3+	0.032		0.029		0.029		0.004		0.005		0.003	
	Al	0.0000		0.000		0.000		0.018		0.027		0.026	
	Cr	0.025		0.025		0.024		0.004		0.002		0.002	
	Ti	0.007		0.006		0.006		0.008		0.010		0.010	
	Mn	0.002		0.001		0.002		0.003		0.003		0.003	
M2	Mg	0.061	(2)	0.067	(2)	0.061	(7)	0.046	(2)	0.059	(4)	0.052	(3)
	Fe	0.092	(1)	0.088	(1)	0.097	(7)	0.104	(2)	0.117	(2)	0.113	(2)
	Ca	0.823		0.825		0.822		0.833		0.799		0.809	
	Mn	0.006		0.003		0.004		0.003		0.004		0.004	
	Na	0.018		0.017		0.017		0.014		0.021		0.021	
	*X*Fe	0.15		0.14		0.15		0.21		0.20		0.19	
	Fs	9		8		9		12		12		12	
	k_D	0.038	(2)	0.036	(2)	0.031	(4)	0.069	(4)	0.062	(5)	0.055	(4)
T_c(°C)		645	(10)	637	(12)	608	(25)	791	(15)	764	(20)	734	(19)

Note: $k_D = [(Fe^{2+}_{M1})(Mg_{M2})/(Fe^{2+}_{M2})(Mg_{M1})]$. The site occupancy values represent atoms per six oxygen atoms. Chemical constraints introduced are based on the chemical analysis (see Table S3).

Table S3 – Chemical analyses (EMPA)

Table S3. Electron microprobe analysis for clinopyroxenes TSC 3.9, 3.12_1, 3.12_2, 3.22, 3.31_1 and 3.31_2 and formula in atoms per formula unit (apfu) based on six oxygen atoms.

	**CPX 3.9** (averaged 10 spots)		**CPX 3.12_1** (averaged 13 spots)		**CPX 3.12_2** (averaged 16 spots)		**CPX 3.22** (averaged 10 spots)		**CPX 3.31_1** (averaged 25 spots)		**CPX 3.31_2** (averaged 38 spots)	
wt%												
SiO2	53.59	(20)	53.72	(13)	53.76	(27)	54.30	(31)	53.74	(55)	53.74	(55)
TiO2	0.25	(2)	0.21	(2)	0.22	(3)	0.30	(3)	0.37	(6)	0.37	(6)
Al2O3	1.18	(7)	1.07	(8)	1.10	(14)	1.27	(8)	1.61	(29)	1.61	(29)
Cr2O3	0.89	(11)	0.86	(7)	0.84	(9)	0.15	(4)	0.08	(5)	0.08	(5)
Fe2O3	0.00	(0)	0.00	(0)	0.00	(0)	0.00	(0)	0.00	(0)	0.00	(0)
FeO	5.31	(20)	4.95	(29)	5.31	(60)	6.85	(28)	8.19	(80)	8.19	(80)
MnO	0.16	(7)	0.13	(2)	0.17	(11)	0.16	(4)	0.19	(3)	0.19	(3)
MgO	18.17	(21)	18.24	(21)	17.98	(47)	16.95	(28)	16.59	(61)	16.59	(61)
CaO	20.86	(16)	21.16	(16)	21.15	(11)	20.98	(19)	20.15	(25)	20.15	(25)
Na2O	0.26	(2)	0.24	(2)	0.24	(2)	0.20	(2)	0.24	(6)	0.24	(6)
K2O	0.00	(0)	0.00	(0)	0.00	(0)	0.00	(0)	0.00	(0)	0.00	(0)
Totale	100.68	(32)	100.58	(18)	100.77	(26)	101.17	(36)	101.18	(53)	101.18	(53)
FeO	4.33	(21)	4.07	(23)	4.39	(65)	6.85	(30)	7.89	(65)	7.89	(65)
Fe2O3	1.09	(11)	0.97	(17)	1.02	(17)	0.00	(18)	0.33	(46)	0.33	(46)
Totale	100.79	(31)	100.68	(19)	100.88	(25)	101.17	(36)	101.21	(54)	101.21	(54)
a.p.f.u												
Si	1.944	(5)	1.949	(3)	1.949	(6)	1.971	(5)	1.958	(13)	1.958	(13)
Ti	0.007	(1)	0.006	(1)	0.006	(1)	0.008	(1)	0.010	(2)	0.010	(2)
Al	0.050	(3)	0.046	(4)	0.047	(6)	0.054	(4)	0.069	(13)	0.069	(13)
Cr	0.026	(3)	0.025	(2)	0.024	(3)	0.004	(1)	0.002	(1)	0.002	(1)
Fe3+	0.030	(3)	0.027	(5)	0.028	(5)	0.000	(5)	0.009	(13)	0.009	(13)
Fe2+	0.131	(6)	0.124	(7)	0.133	(20)	0.208	(9)	0.240	(21)	0.240	(21)
Mn	0.005	(2)	0.004	(1)	0.005	(3)	0.005	(1)	0.006	(1)	0.006	(1)
Mg	0.983	(10)	0.987	(11)	0.972	(22)	0.917	(13)	0.901	(30)	0.901	(30)
Ca	0.811	(7)	0.822	(6)	0.822	(4)	0.816	(7)	0.787	(9)	0.787	(9)
Na	0.019	(1)	0.017	(2)	0.017	(1)	0.014	(2)	0.017	(4)	0.017	(4)
K	0.000	(0)	0.000	(0)	0.000	(0)	0.000	(0)	0.000	(0)	0.000	(0)
Totale	4.005	(3)	4.006	(3)	4.004	(2)	3.998	(3)	4.000	(2)	4.000	(2)
Al IV	0.050	(3)	0.046	(3)	0.047	(6)	0.029	(5)	0.042	(13)	0.042	(13)
Al VI	0.000	(1)	0.000	(1)	0.000	(0)	0.025	(5)	0.027	(6)	0.027	(6)
m.a.n*	33.29	(15)	33.21	(11)	33.32	(32)	33.63	(15)	34.00	(38)	34.00	(38)

m.a.n.*: calculated total mean atomic number for *M*1, *M*2 and *M*21 sites, in electrons per formula unit. Standard deviations are given in parentheses.

Table S4 – Physical properties of the lithological units

	**ρ** (g/cm^3)	**cp** (J/Kg/K)	**Th.cond** (W m^{-1} K^{-1})
**Peridotite**	3.19	1000	3
**Pyroxenite**	3.2	750	4.3
**Gabbro-Pyroxenite**	2.9	500	2.5

Table S4. Physical properties of the three lithologies (density, heat capacity and thermal conductivity).
